# Oro-Respiratory Dysbiosis and Its Modulatory Effect on Lung Mucosal Toxicity during Exposure or Co-Exposure to Carbon Nanotubes and Cigarette Smoke

**DOI:** 10.3390/nano14030314

**Published:** 2024-02-04

**Authors:** Brijesh Yadav, Sukanta S. Bhattacharya, Lauren Rosen, Ravinder Nagpal, Hariom Yadav, Jagjit S. Yadav

**Affiliations:** 1 Pulmonary Pathogenesis and Immunotoxicology Laboratory, Department of Environmental and Public Health Sciences, University of Cincinnati College of Medicine, Cincinnati, OH 45267-0056, USA; brijeshsgpgims@gmail.com (B.Y.);; 2 Department of Pathology and Laboratory Medicine, University of Cincinnati, UC Health University Hospital Laboratory Medicine Building, Suite 110234 Goodman Street, Cincinnati, OH 45219-0533, USA; 3 Department of Nutrition and Integrative Physiology, Florida State University, Tallahassee, FL 32306, USA; 4 USF Center for Microbiome Research, Department of Neurosurgery and Brain Repair, Internal Medicine-Digestive Diseases and Nutrition, University of South Florida, Tampa, FL 33613, USA

**Keywords:** carbon nanotubes, cigarette smoke extract, lung microbiome, oral microbiome, nasal microbiome

## Abstract

The oro-respiratory microbiome is impacted by inhalable exposures such as smoking and has been associated with respiratory health conditions. However, the effect of emerging toxicants, particularly engineered nanoparticles, alone or in co-exposure with smoking, is poorly understood. Here, we investigated the impact of sub-chronic exposure to carbon nanotube (CNT) particles, cigarette smoke extract (CSE), and their combination. The oral, nasal, and lung microbiomes were characterized using 16S rRNA-based metagenomics. The exposures caused the following shifts in lung microbiota: CNT led to a change from Proteobacteria and Bacteroidetes to Firmicutes and Tenericutes; CSE caused a shift from Proteobacteria to Bacteroidetes; and co-exposure (CNT+CSE) had a mixed effect, maintaining higher numbers of Bacteroidetes (due to the CNT effect) and Tenericutes (due to the CSE effect) compared to the control group. Oral microbiome analysis revealed an abundance of the following genera: Acinetobacter (CNT), Staphylococcus, Aggregatibacter, Allobaculum, and Streptococcus (CSE), and Alkalibacterium (CNT+CSE). These proinflammatory microbial shifts correlated with changes in the relative expression of lung mucosal homeostasis/defense proteins, viz., aquaporin 1 (AQP-1), surfactant protein A (SP-A), mucin 5b (MUC5B), and IgA. Microbiota depletion reversed these perturbations, albeit to a varying extent, confirming the modulatory role of oro-respiratory dysbiosis in lung mucosal toxicity. This is the first demonstration of specific oro-respiratory microbiome constituents as potential modifiers of toxicant effects in exposed lungs.

## 1. Introduction

Engineered nanomaterials (NMs) offer immense potential for myriad applications in several sectors, which has led to an escalation of their production over the last decade. Carbon nanotubes (CNTs) in particular are high-volume engineered nanomaterials that find wide-ranging applications in diverse sectors, including manufacturing, medicine, and the environment [1]. However, their perceived impact on human health and the environment has led to contemplation of whether the risks largely outweigh their benefits. Indeed, the risk of human exposure to CNTs in occupational settings as well as through the environment during the product life cycle of these materials is high since CNTs can become airborne and persist as aerosols. CNT nanoparticles are therefore considered a potential human respiratory health hazard. The past decade has seen several epidemiological and animal studies highlighting the toxicity and respiratory effects of CNTs [2]. Published rodent model studies including our own indicate their potential to induce lung inflammatory episodes causing acute response and chronic toxicologic immunopathology characterized by inflammation, hyperplasia, and granulomatous lesions [3,4,5,6]. Our sub-chronic mouse model studies have shown that hyperplasia in response to inhaled CNTs preferentially involves the proliferation of type 2 pneumocytes (T2Ps), also known as alveolar type II epithelial cells lining the alveoli, conceivably to heal the injury and maintain epithelial integrity [5]. CNT toxicity may be even more critical in workplaces with smokers in the workforce because of co-exposure to tobacco smoke constituents. While the underlying mechanisms of the immunotoxic effects of these nanoparticles and toxicants are emerging [7], their impact on the microbiome of the air passages and its consequences are poorly understood.

The role of microbiomes in human health is becoming increasingly apparent with the gradual revelation of data in the area of disease–microbiome crosstalk [8,9,10,11]. It is now known that microbiome balance within the body (eubiosis) is critical for a healthy human body. This status may be altered, leading to an imbalance (dysbiosis), due to several factors, such as sedentary lifestyle, food habits, and exposure to pollutants. These may include voluntary (tobacco smoke) and involuntary (air pollution, occupational exposures) pollutants, among others. Dysbiosis involves perturbations in microbial abundance and diversity (measured in terms of diversity indices such as Chao1 and Shannon) and has been linked with the onset of several diseases, though the underlying mechanisms remain poorly understood. Often, it has been speculated that an altered microbiome resulting from respiratory exposure to toxicants, such as smoke and air pollution, may be responsible, at least in part, for the onset of the associated pathologies/diseases. The microbiome segment that has been mostly investigated in such a toxicant exposure context is the gut microbiome. However, the oro-respiratory microbiome (microbiota in upper and lower parts of the respiratory tract, including the oral cavity), being at the environment–respiratory system interface, may be critical in mediating host response to inhalable toxicants via direct and indirect interactions [12,13,14,15]. For instance, it has been observed that in the airway microbiome, the Gamma-proteobacteria group benefits from the inflammatory lung condition, while encoding molecular components promoting inflammation. In contrast, genera such as Prevotella may participate in the immunologic homeostasis of the airways [16,17]. These findings are not only indicative of the airway microbiome playing a critical role in lung immunopathology but also suggest a quite different immunogenic response of the airway microbiome compared to the gut microbiome. Taken together, this scientific premise has triggered increased research interest in microbial dysbiosis in the lung and upper parts of the respiratory system and their role in understanding the mechanisms underlying the toxicity of inhalable pollutants.

In human respiratory diseases, there has been increasing evidence of the role of the lung microbiome in inducing immune responses and vice versa [16,18]. For instance, the role of lung microbiota has been implicated in respiratory immune diseases, particularly asthma [19]. Likewise, tobacco smoke, which is the underlying cause of chronic obstructive pulmonary disease (COPD) and other lung diseases, has been known to affect both oral microbiota [20,21] and lung microbiota [16,22,23,24,25]. However, such information is lacking on oro-respiratory microbiota perturbations during CNT exposures and associated lung toxicity.

Lung homeostasis is maintained by critical mucosal proteins [26]. For example, surfactant proteins such as SP-A are required for the lubrication of the alveolar lining for normal lung function and the opsonization of microbes. Aquaporins (AQPs) are water channel proteins, of which AQP-1 is required for water transport across membranes, particularly of endothelial cells in the lungs. Mucin protein secreted by goblet cells is a critical component of the mucociliary escalator in the lung and is required for inhibiting microbial adherence to epithelial cells, mucosal lubrication, and surface tension reduction in the lung. Immunoglobulin A (IgA) is a predominant antibody isotype in mucosal surfaces where it confers protection from invading pathogens while being tolerant to commensals (which are considered as its inducers) and is a potential driver of microbiota homeostasis [27]. Lung exposure to pathogens and toxicants, such as cigarette smoke and associated respiratory diseases, disrupts lung mucosal homeostasis as well as the lung microbiome; however, the causal role of the microbiome in such mucosal disruptions implicating the mucosal proteins is unclear. Such interactions are particularly unclear in lung exposure to engineered nanoparticles.

In view of the above scientific premise, our hypothesis is that CNT nanoparticles, alone or in combination with cigarette smoke (frequently co-occurring exposures), may cause perturbations (dysbiosis) in the oro-respiratory microbiome, which may play a modifying role in inducing changes in mucosal homeostasis in exposed lungs. To test this in the current study, our overall objective was to investigate the oro-respiratory microbiome in relation to lung mucosal responses employing our previously established CNT sub-chronic exposure mouse model using normal (microbiota-intact) and antibiotic-treated (microbiota-depleted) mice. Considering that smoking is a common confounder in lung exposures, we undertook an investigation of the individual and combined effect of CNTs and cigarette smoke extract (CSE) using microbiota-intact (normal) mice or microbiota-depleted mice to understand any continuum or synergy between these two particulate exposure types. To our knowledge, this is the first report on the effects of CNT nanoparticles (alone or in combination with cigarette smoke) on the oro-respiratory microbiome and the latter’s modulatory role in CNT toxicity in exposed lungs.

## 2. Materials and Methods

### 2.1. CNT Preparation and Characterization

Multi-wall carbon nanotubes (MWCNTs, referred to as CNTs in this report) of high purity were obtained in a dry bulk powder form from Baytubes (Leverkusen, Germany) and physiochemically characterized and processed as described in our previous report [5,7]. A CNT suspension was prepared in 1% Pluronic solution made in PBS for use in mice exposures.

### 2.2. CSE Preparation

Cigarette smoke extract (CSE) was prepared from reference cigarettes (Code 3R4F), obtained from the Center for Tobacco Reference Products, Kentucky Tobacco Research & Development Center, Lexington, KY, USA, using an in-house protocol, as reported earlier [7]. The resulting extract was collected aseptically and stored in airtight vials at 4 °C until used.

### 2.3. Animals, Microbiota Depletion, and Toxicant Exposures

Six-week-old male mice were generated by in-house breeding using C57BL/6J breeding mice pairs purchased from The Jackson Laboratory (JAX). A cohort of microbiota-depleted mice (designated as ABX mice) was generated by administration of antibiotics for 2 weeks, per our published protocol [7]. Age-matched (8-week-old) microbiota-intact (WT) mice and the microbiota-depleted (ABX) mice groups were then divided into four exposure cohorts to test the impact of the following exposures: (a) vehicle (1% Pluronic F127 in PBS) as the control group, (b) CNT, (c) CSE, and (d) a combination of CNT and CSE. Oropharyngeal administration was carried out for all exposure treatments. For CNT treatment, a single dose (2.8 mg kg^−1^ body weight) of CNT suspension was administered on day one and the treated mice were housed normally for 4 weeks. For the CSE treatment, a daily dose (30 µL/mouse) of CSE was administered for 4 weeks. For the co-exposure regime, the mice were administered the above-described CNT and CSE dose regimens for a total exposure period of 4 weeks. The animal protocol (#06-12-06-01) for procedures used in this study was approved by the University of Cincinnati’s Institutional Animal Care and Use Committee (IACUC).

### 2.4. Organ Harvesting and Microbial Cell Recovery

All experimental mice were sacrificed by intraperitoneal injection of Euthasol^®^ (Butler-Schein, Dublin, OH, USA) per the approved IACUC protocol. The lungs were lavaged to recover bronchoalveolar lavage (BAL) fluid and the tissue was harvested as per the protocols described in our earlier report [7,26]. For oral and nasal lavaging, a small nick was made at the pharynx exposing both the nasal and oral cavities. The two cavities were rinsed with 1 mL sterile PBS each and the resulting lavage was centrifuged in succession at 1000 RPM/5 min and 12,000 RPM/15 min at room temperature to differentially pellet the mouse cells and the microorganisms, respectively. The microbial pellet was used for microbiome analysis and the supernatants were stored immediately at −80 °C for subsequent measurements.

### 2.5. Microbial DNA Isolation and Nextgen Sequencing

The microbial pellets obtained from the BAL fluid, oral lavage, and nasal lavage samples were processed for isolation of total microbial DNA using Qiagen’s DNeasy Blood and Tissue kit (Qiagen Inc., Germantown, MD, USA), following the manufacturer’s protocol with modifications. Briefly, the microbial pellet was lysed using an optimized lysis buffer (20 mM Tris-HCl, pH 8.0, 2 mM Sodium EDTA, 1.2% Triton X-100, 20 mg/mL Lysozyme added immediately before use, 40 IU/mL Mutanolysin) at 37 °C for 30 min in conjunction with bead beating. The buffer AL of the kit was then added to the crude lysate followed by purification of the microbial DNA using the DNeasy Mini Spin column per the manufacturer’s protocol. To check any background contamination, microbial DNA was extracted from the blank microfuge tubes using the same reagents and conditions. The DNA extract was stored (−80 °C) for subsequent analysis. The *16S rRNA* gene sequencing was performed using the MiSeq V2 500 cycle kit on an Illumina platform as described earlier [28], at the DNA Core of the Cincinnati Children’s Hospital Medical Center (CCHMC). Bioinformatic analysis of the data involved FASTQ file generation followed by the use of the QIIME program, demultiplexing, reformatting, dereplication and operational taxonomic unit (OTU) clustering, taxonomic assignment, and OTU table creation followed by analysis for alpha and beta diversity [29]. Linear discrimination analysis (LefSe) was performed using a Galaxy module based on a 3.0 LDA score threshold value (http://huttenhower.sph.harvard.edu/galaxy, accessed on 1 June 2020).

### 2.6. RNA Isolation

Mouse lung tissue was processed for total RNA extraction using a TRI reagent kit following the manufacturer’s protocol. Briefly, 150 mg of the tissue was homogenized in 1 mL of TRI reagent solution on ice using a tissue homogenizer (Biospec Variable speed Tissue Tearor, model no. 985370). The homogenate was subjected to phase separation by treatment with 100 µL of 1-bromo-3-chloropropane (BCP) for 5 min at room temperature, followed by centrifugation at 12,000 RPM for 12 min at 4 °C. The aqueous phase was subjected to a column-based protocol using a RNeasy mini kit (Qiagen) for RNA purification. The quality and concentration of the resulting RNA preparation were estimated using Nanodrop (Nunc Nanodrop, Thermofisher, Waltham, MA, USA).

### 2.7. qRT-PCR for Gene Expression Analysis

Gene expression analysis was performed based on gene-specific qRT-PCR using a Brilliant III SYBER Green Master Mix one-step kit (Agilent Technologies, Santa Clara, CA, USA) and real-time PCR platform ABI 7500 (Applied Biosystems, Waltham, MA, USA). The housekeeping gene hypoxanthine phosphoribosyl transferase (HPRT) was used as an internal control. Gene-specific primers were used for transcriptional analysis of the following targets: aquaporin 1 (AQP-1), surfactant protein A (SP-A), and mucin 5b (MUC5B). The primers were used from published sources (see Supplementary Table S1) [26]. One-step qRT-PCR reaction for each gene target was performed using an ABI 7500 thermocycler (Applied Biosystems) using the reaction conditions described in our earlier report [26]. Fold change (FC) in target gene expression in the sample relative to the control was calculated by the 2^−δδCT^ method [30]. The calculation was based on the following formula: Fold Change (FC) = 2^−[CT(TG)-CT(HKG)]^ treated sample − 2^−[CT(TG)-CT(HKG)]^ vehicle control. Regulation of gene expression was interpreted as follows: FC = 1, no change in gene expression; FC > 1, upregulation; FC < 1, downregulation.

### 2.8. IgA Estimation in BAL Fluid

Immunoglobin-A (IgA) was measured in the BAL fluid using an IgA Mouse ELISA kit (cat. no. 88-50450, Thermofisher Scientific USA) following the manufacturer’s protocol. Briefly, the ELISA plate was coated with capture antibody (100 µL) overnight at 4 °C. Wells were aspirated and washed with wash buffer (400 µL/well) and blocked with blocking buffer (250 µL/well). The wells were washed and loaded with the standard or sample (10 µL diluted in 90 µL of assay buffer) and incubated for 2 h at room temperature on the shaker. The wells were washed again, and a detection antibody (100 µL/well) was added. Following another wash, a substrate solution (100 µL/well) was added and incubated for 15 min. The reaction was stopped with 100 µL of stop solution and absorbance was measured at 450 nm. The IgA concentration was calculated relative to the standard concentration. The minimum detection limit of the IgA kit was 0.39 ng/mL.

### 2.9. Histopathological Analysis of Lung Tissue

The formalin-fixed lung tissues (*n* = 2 mice) for each treatment type (CNT, CSE, and CNT+CSE) were stained with hematoxylin and eosin (H&E) staining at the CCHMC Histology core facility. A blinded histopathological analysis of the stained sections was carried out using an Olympus BX53 microscope equipped with Olympus DP22 and the software imaging system cellSens standard 2.2 (Olympus Lifescience, Waltham, MA, USA). The endpoints analyzed include alveolar type II pneumocyte (T2P) hyperplasia and bronchoalveolar (mixed-cell) hyperplasia in terms of location, presence or absence, and severity. The observations were scored on a scale of 0 to 4 where 0 is absent, 1 is minimal, 2 is mild, 3 is moderate, and 4 represents severe. Each slide was analyzed for five adjacent areas and representative images were captured for presentation.

### 2.10. Statistical Analyses

All statistical analyses were performed using GraphPad Prism 8.0.1 (La Jolla, CA, USA). The results were expressed as mean ± standard error (SE) and further statistical significance of the difference between groups was calculated by using an unpaired *t*-test with Welch correction. Statistical significance was determined with a *p*-value ≤ 0.05 representing significance and the lower values *p* ≤ 0.01 and *p* ≤ 0.001 representing highly significant differences. The level of significance was denoted using asterisks as follows: (*) if *p* ≤ 0.05, (**) if *p* ≤ 0.01, and (***) if *p* ≤ 0.001.

## 3. Results

### 3.1. Toxicant Exposure and Co-Exposure Led to Oro-Respiratory Microbiota Dysbiosis in WT Mice

Microbiome analyses in the lung, nasal cavity, and oral cavity showed distinct shifts in the overall microbial community composition and differential abundance of specific bacterial taxa between the control mice and the CNT- and/or CSE-exposed mice, as detailed below.

#### 3.1.1. Lung Microbiota Dysbiosis

**Figure 1:**
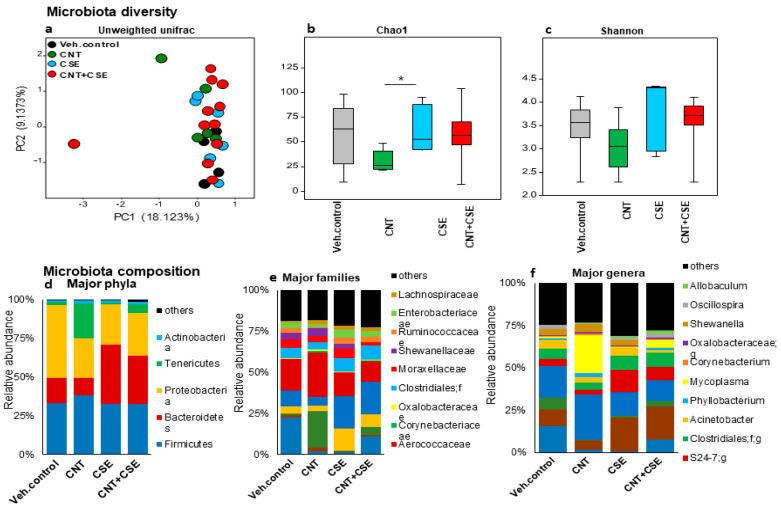
Lung microbiota diversity and compositional changes in mice in response to respiratory toxicants. The toxicants, animal exposure conditions, and lung microbiota DNA analyses are described in the Materials and Methods (Section 2). The top part of the figure depicts microbiota diversity showing differential microbiota clustering for beta diversity based on principal component analysis (**a**) and alpha diversity based on Chao1 index (**b**) and Shannon index (**c**), between the control and treated groups. The bottom part of the figure depicts microbiota composition at the phylum (**d**), family (**e**), and genus (**f**) level showing distinct differences in the CNT, CSE, and CNT+CSE groups compared to the control group. The differences in diversity and composition suggest definitive effects of the toxicant exposures.

**Figure 2:**
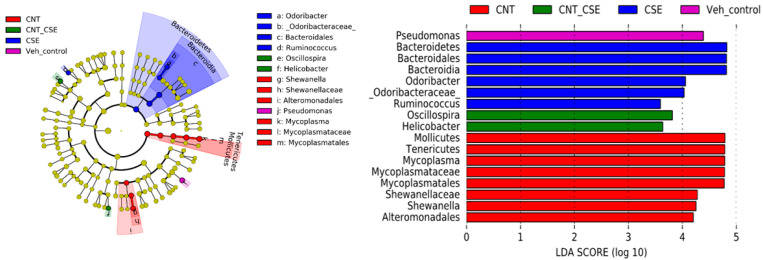
Dominant operational taxonomical units (OTUs) of the lung microbiome in mice exposed to respiratory toxicants. The toxicants, animal exposure conditions, and lung microbiota DNA analyses are described in the Materials and Methods (Section 2). The LEfSe analysis shows a higher proportion of Mollicutes, *Mycoplasma*, *Tenericutes*, *Shewanella*, and Altermonadales in the CNT group; Bacteroidetes, *Odoribacter*, and *Ruminococcus* in the CSE group; and *Oscillospira* and *Helicobacter* in the CNT+CSE group.

The analysis of lung microbiota showed differential microbiota clustering, particularly when holistically comparing control and treated samples for diversity (Figure 1), suggesting definitive effects of exposures. At the phylum level, the CNT group had a reduced abundance of Bacteroidetes and Proteobacteria while having an increased abundance of Firmicutes and Tenericutes (Figure 1). Accordingly, the microbiota composition at the genus level was found to be distinctly different in the CNT, CSE, and CNT+CSE groups compared to the control group. Comparison of these bacterial taxa by LEfSe analysis revealed a significantly higher proportion of Mollicutes, *Mycoplasma*, *Tenericutes*, *Shewanella*, and Altermonadales in the CNT group; Bacteroidetes, *Odoribacter*, and *Ruminococcus* in the CSE group; and *Oscillospira* and *Helicobacter* in the CNT+CSE group (Figure 2). The abundance of *Pseudomonas* was significantly lower in all three treated groups compared to the control group (Figure 2).

#### 3.1.2. Oral Microbiota Dysbiosis

**Figure 3:**
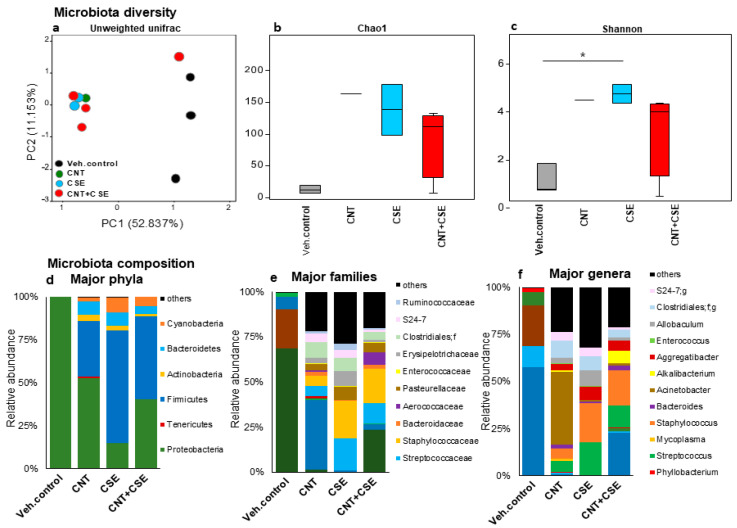
Oral microbiota diversity and compositional changes in mice in response to respiratory toxicants. The toxicants, animal exposure conditions, and oral microbiota DNA analyses are described in the Materials and Methods (Section 2). The top part of the figure depicts oral microbiota diversity showing differential microbiota clustering between the control and treated groups for beta diversity based on principal component analysis (**a**) and alpha diversity based on Chao1 index (**b**) and Shannon index (**c**). The bottom part of the figure depicts oral microbiota compositional differences among the treatment groups at phylum (**d**), family (**e**), and genus (**f**) levels. At the genus level, the CNT group had a higher abundance of *Acinetobacter*, the CSE group had a higher abundance of *Staphylococcus*, *Aggregatibacter*, *Allobaculum*, and *Streptococcus*, and the CNT+CSE group had *Alkalibacterium* abundance.

Interestingly, a pattern similar to that in the lung microbiota was seen in the oral microbiota wherein the CNT, CSE, and CNT+CSE groups demonstrated distinct clusters compared to the control group (Figure 3). At the phylum level, Proteobacteria abundance was reduced, while Firmicutes, Cyanobacteria, and Bacteroidetes were increased in all three toxicant treatment groups. In addition, at the genus level, the CNT group had a higher abundance of *Acinetobacter* while the CSE group had a higher abundance of *Staphylococcus*, *Aggregatibacter*, *Allobaculum*, and *Streptococcus*, and the CNT+CSE group had *Alkalibacterium* abundance (Figure 3).

#### 3.1.3. Nasal Microbiota Dysbiosis

**Figure 4:**
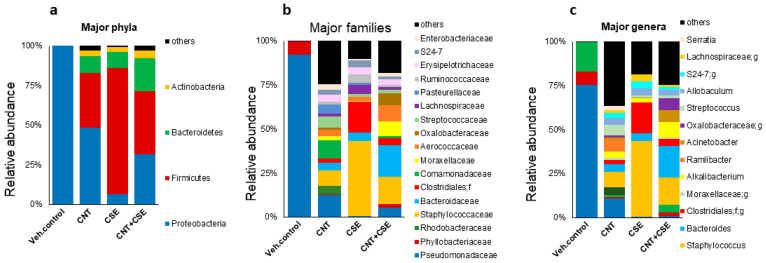
Compositional changes in mouse nasal microbiota at various phylogenetic levels in response to different respiratory toxicants. The toxicants, animal exposure conditions, and nasal microbiota analyses are described in the Materials and Methods (Section 2). Nasal microbiome compositional differences among the treatment groups are shown at phylum (**a**), family (**b**), and genus (**c**) levels. The analysis revealed reduced Proteobacteria and increased Firmicutes in the CNT, CSE, and CNT+CSE groups compared to the control group.

Although a sufficient sample size could not be acquired for nasal specimens, the analysis of nasal microbiota demonstrated a similar pattern to that seen for the oral microbiota, i.e., reduced Proteobacteria and increased Firmicutes in the CNT, CSE, and CNT+CSE groups compared to the control group (Figure 4). The three treatment groups segregated well from the control group and showed a trend towards high beta diversity but low alpha diversity differences among their microbiota profiles.

Taken together, the above-described microbiome data clearly indicated that the respiratory exposure and co-exposure to the examined particulates, CNTs in particular, result in a dysbiosis of lung, oral, and nasal mucosal microflora characterized by a shift in microbial community composition and altered abundance of several bacterial taxa.

### 3.2. Microbiota Depletion Differentially Regulated the Lung Mucosal Homeostasis/Defense Proteins

We investigated the transcriptional regulation of mucin (MUC5B), surfactant protein-A (SP-A), and aquaporin-1 (AQP-1) based on gene expression analysis. Regulation of these lung-function-related proteins in normal (WT) microbiota-intact mice when exposed to CNT, CSE, or CNT+CSE was reported in our previous report [26]. Here, we compared antibiotic-treated (ABX) microbiota-depleted mice with the WT microbiota-intact mice (as comparison control) to understand the modulatory role of microbiota in toxicant-induced lung damage/injury. Though the baseline in ABX background mice was somewhat higher than in the WT background mice, the overall outcomes in the two backgrounds were exposure-dependent, confirming the driver role of toxicant–microbiota interactions.

#### 3.2.1. Microbiota Depletion Upregulated Mucin (MUC5B) Gene Expression

**Figure 5:**
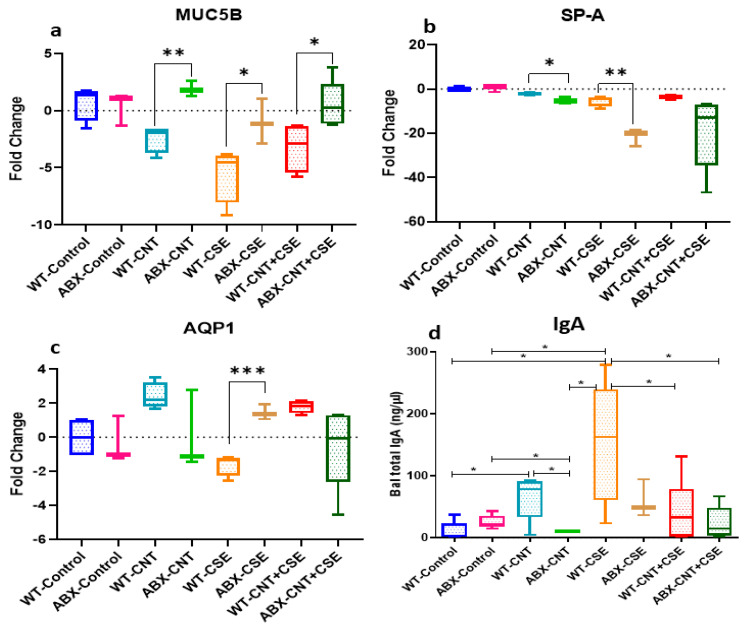
Mucosal defense and homeostasis proteins in the exposed lungs from microbiota-depleted (ABX) mice relative to microbiota-intact (normal) mice. The toxicants and animal exposure conditions are described in the Materials and Methods (Section 2). The gene expression analysis for the following individual proteins measured in the lung tissue showed differential expression among the treatment groups: (**a**) Mucin 5b (MUC5B); (**b**) Surfactant protein-A (SP-A); (**c**) Aquaporin-1 (AQP-1). (**d**) Immunoglobulin A (IgA) level measured in the bronchoalveolar lavage (Bal) fluid was lower in microbiota-depleted mice. Note that the WT data presented here for comparison are leveraged from our earlier open access publication (Reference [26] published by “Frontiers”). Statistical comparisons between treatments are represented in terms of the level of significance (*p*-value) denoted using asterisks, as follows: (*) if *p* ≤ 0.05, (**) if *p* ≤ 0.01, and (***) if *p* ≤ 0.001.

This mucin protein isoform, required for lung airway lubrication/rheology and barrier function, showed relative upregulation in microbiota-depleted (ABX) mice in the three exposure groups, viz., CNT (*p* ≤ 0.01), CSE (*p* ≤ 0.05), and CNT+CSE (*p* ≤ 0.05), compared to the normal (WT) background (Figure 5a).

#### 3.2.2. Surfactant Protein-A (SP-A) Was Downregulated in Microbiota-Depleted Mice

Lung surfactant protein A showed relative downregulation in the CNT (*p* ≤ 0.05), CSE (*p* ≤ 0.01), and CNT+CSE exposure groups in the ABX background compared to the WT background (Figure 5b).

#### 3.2.3. Microbiota Depletion Exerted a Differential Effect on Aquaporin-1 (AQP-1) Gene Expression

AQP-1 expression was relatively upregulated in the microbiota-depleted (ABX) background in the CSE exposure group (*p* ≤ 0.001) but showed a downregulation trend in the CNT and CNT+CSE groups compared to the WT background (Figure 5c).

#### 3.2.4. Microbiota Depletion Reduced Total Immunoglobulin A (IgA) Secretion in Lung

The total IgA level in the BAL fluid was significantly high in the CNT and CSE groups compared to the vehicle control group (*p* < 0.05) in the WT background mice. In a pairwise comparison between WT (microbiota-normal) and ABX (microbiota-depleted) mice, the IgA level was relatively lower in the microbiota-depleted group across all three toxicant treatment groups.

Taken together, the above results suggested that microbial dysbiosis due to toxicant exposures differentially impacts the regulation of key lung homeostasis or defense proteins under different toxicant exposure conditions (Figure 5d).

### 3.3. Microbiota Dysbiosis Modulated the CNT- and/or CSE-Induced Lung Pathology

**Figure 6:**
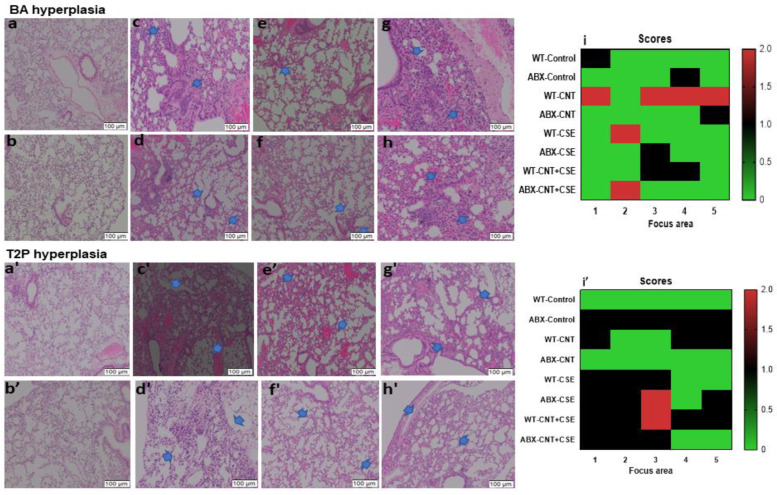
Hyperplasia in the exposed lungs of microbiota-intact (normal) versus microbiota-depleted (ABX) mice subjected to different respiratory toxicants. The hyperplasia changes are shown by arrows in the H&E-stained images. The toxicants and animal exposure conditions are described in the Materials and Methods (Section 2). Bronchoalveolar (BA) hyperplasia is presented in the top part of the figure as follows: upper panels—(**a**) WT-Control; (**c**) WT-CNT; (**e**) WT-CSE; (**g**) WT-CNT+CSE; lower panels—(**b**) ABX-Control; (**d**) ABX+CNT; (**f**) ABX+CSE; (**h**) ABX+CNT+CSE. (**i**) Heat map showing the effects of different exposures. Type 2 pneumocyte (T2P) hyperplasia, also known as alveolar Type2 epithelial cell hyperplasia, is presented in the bottom part of the figure as follows: upper panels—(**a’**) WT-Control; (**c’**) WT-CNT; (**e’**) WT-CSE; (**g’**) WT-CNT+CSE; lower panels—(**b’**) ABX-Control; (**d’**) ABX+CNT; (**f’**) ABX+CSE; (**h’**) ABX+CNT+CSE. (**i’**) Heat map showing the effects of different exposures. The *x*-axis numbers 1–5 in panel (**i**) and (**i’**) represent the five different focus areas analyzed per slide. The *y*-axis numbers 0, 0.5, 1.0,1.5, and 2.0 in panel (**i**) and (**i’**) represent the injury scores on a 0 to 4 scale, with 0 being normal and 4 being severe. Microbiota depletion resulted in a lower grade of hyperplasia (BA and T2P) in either CNT- or CSE-exposed mice.

Vehicle control mice showed no sign of any deformation in the lung for any of the histological markers. Normal mice (WT) treated with CNT alone showed type II pneumocytes (T2P) hyperplasia and bronchoalveolar (BA) hyperplasia. However, in ABX-CNT (microbiota-depleted CNT-treated) mice, lower grades of T2P hyperplasia and BA hyperplasia were observed. ABX-CSE mice showed mild grade T2P hyperplasia and BA hyperplasia. Similarly, in normal CNT+CSE and ABX-CNT+CSE treated mice, the patchy T2P and BA hyperplasia were observed but the extent of these changes was lower in ABX-treated mice (Figure 6).

## 4. Discussion

There is emerging interest in understanding the role of microbiomes in environmental-exposure-associated lung diseases such as asthma and COPD, which are caused by involuntary exposures to toxicants like CNTs and/or voluntary exposure to toxicants such as smoking, and other environmental triggers. However, the majority of such studies, including our own, on microbiome–toxicant pathology interactions have focused on the gut microbiome [12,13,14,15,26] and little is known about the role of the oral and respiratory microbiomes. While cigarette smoking has been shown to modulate human oral and lung microbiomes [22,23,24] such evidence is lacking for other toxic particulate types, particularly for carbon nanotubes, the high-volume engineered nanoparticles that are known to cause lung damage and pathological conditions [4,5,6]. In this context, our most recent studies have shown that respiratory exposure to CNTs can remotely induce dysbiosis in gut microbiota [7]. In the current study, the question of whether respiratory exposure to CNT particles is responsible for microbiome perturbations in the local organ (respiratory tract) was investigated using the CNT sub-chronic respiratory exposure mouse model. An additional query was to investigate whether the impact is primarily confined to the main lung or if it extends throughout the entire respiratory tract, including the upper parts (nasal or oral) involved in the respiration process.

While the health effects of inhaled carbon nanoparticles, particularly CNTs, have attracted much attention, little is known about their synergy with commonly inhaled particulates, such as cigarette smoke. The current study was designed to primarily understand the effects of respiratory exposure as well as co-exposure to CNT particles and cigarette smoke constituents on oro-respiratory microbiota and whether the microbiota is a critical regulator of lung toxicity in these exposures. Such a regulatory role of the microbiota in the exposed lungs was investigated by comparing mice with intact (normal WT) lung microbiota and those with antibiotic-depleted (ABX) microbiota.

### 4.1. Dysbiosis of Oro-Respiratory Microbiota Due to Respiratory Exposure to CNT and/or Tobacco Smoke Constituents

Different treatment groups of mice were analyzed for microbiota composition in different segments of the oro-respiratory system. We observed an overlap of Proteobacteria among the three microbiomes (lung, oral, and nasal). Additionally, the lungs harbored the microbiome phyla Bacteroidetes, Firmicutes, and Actinobacteria. This suggested the prevalence of both overlapping and niche-specific colonization of individual segments of the oro-respiratory tract. In terms of factors perturbing the oro-respiratory microbiota, inhalation exposures to smoke and air pollution have been shown to cause dysbiosis of both the lung and oral microbiota [22,31]. Such dysbiosis bears significance considering that oral microbiota has been linked with esophageal cancer [32] and chronic kidney diseases [33] and there is emerging evidence on the role of lung microbiota in lung health and disease [16,18].

#### 4.1.1. Lung Microbiota

In the lung, the microbiota is considered to be one of the factors contributing to the first line of defense and is required for normal lung function [34]. The lung being in continuity with the upper respiratory tract, it is unclear whether the nasal microbiota is the potential source for the lung microbiota. Comparative analysis of the control and treated mice groups showed that Proteobacteria are the overlapping major phylum in the nasal and oral microbiomes; this phylum formed a dominant fraction (>99%) of these microbiotas in the control mice. Interestingly, Proteobacteria constituted a large proportion (46.9%) of the total lung microbiome, which also contained Bacteroidetes (16.7%), Firmicutes (32.8%), and Actinobacteria (1.1%); this implied a selective overlap between the lung microbiome profile and those of the oral and nasal microbiome (Figure 1, Figure 2, Figure 3 and Figure 4). Although a similar lung microbiome profile was observed in other mouse studies, the composition is highly variable as it may depend on shipment, vendor, cage, and surrounding environmental factors [35,36,37]. This implies that the source of lung microbiota may be environmental and can be manipulated by specific external factors.

Our observations that the predominant phyla in the mouse lung microbiota are Proteobacteria, Firmicutes, and Bacteroidetes are similar to the findings in human studies on asthma patients [38,39]. Higher levels of the phylum Proteobacteria (Gram-negative bacteria) have been positively correlated with Th17 cell development and recruitment of eosinophils and neutrophils in the lung [40]. Similarly, higher levels of the phylum Firmicutes (Gram-positive bacteria) and the genus *Lactobacillus* were found to be associated with severe asthma [41]. Higher Bacteroidetes abundance promoted programmed death ligand (PD-L1) restricted T regulatory cells and limited the degree of inflammation in the lung after antigen exposure [42]. While exposure to CNTs led to a shift in lung microbiota from Proteobacteria (also known as *Pseudomonadota*) and Bacteroidetes to Firmicutes (now known as *Bacillota*) and Tenericutes (renamed as *Mycoplasmatota*), the CSE exposure shifted it from Proteobacteria to Bacteroidetes. The co-exposure (CNT+CSE) showed a mixed effect on lung microbiota, maintaining higher numbers of Bacteroidetes (CNT effect) and Tenericutes (CSE effect) compared to the control group (Figure 1). While many species of *Mycoplasma* are known to be harmless, species such as *M. pneumoniae* have been associated with lung diseases, such as walking pneumoniae. Cell-free extracts of *M. pneumoniae* have been shown to trigger immunogenic responses in murine models, which included IL-6, TNF-α, NF-κB, and p38 and PI3K-linked pro-inflammatory signals [43]. Increased Bacteroidetes and Prevotella have been shown to secrete extracellular vesicles that were linked with Th17 cell development and neutrophil recruitment leading to pulmonary fibrosis [34]. Other murine studies have shown shifting of microbiota early in infancy from Gamma-proteobacteria and Firmicutes to Bacteroidetes, leading to PD-L1 dependent anti-inflammatory Treg cell development and attenuation of the Th2 cell response for pollen allergy [42].

#### 4.1.2. Oral Microbiota

Cigarette smoking has been associated with the depletion of oral microbiota in smokers characterized by decreased alpha diversity in buccal mucosa but not in other oral sampling sites [44]. However, in the current mouse model, we noticed an increase in alpha diversity (Shannon index) in the oral microbiome in the CSE-exposed mice. Surfactant proteins (SPs) regulate the colonization of microbiota at mucosal surfaces, such as the oral, lung, and gastrointestinal systems, and also modulate the innate immune response [45]. A study on female smokers with oral lesions reported a differential quantitative effect of smoking and SP-A expression on 53 oral microbiome species, where a higher SP-A expression was associated with decreased oral microbial diversity. The smoker group had a lower salivary SP-A level and a higher abundance of *Corynebacterium argentoratense* [9]. In the current mouse study, we found a decreased SP-A mRNA transcript expression in mice exposed to CSE, CNT, or CNT+CSE. However, both the oral and nasal microbiomes in the CNT, CSE, and co-exposed mice showed an abundance of Proteobacteria and Firmicutes. The Firmicutes abundance was >2-fold higher in the CSE-treated group compared to the CNT-treated group or the CNT+CSE co-treated group mice [42]. An increase in Firmicutes members has been associated with declining lung function in COPD patients [31].

The human lung microbiome in smokers showed an increased proportion of pathogenic species *Acinetobacter* and *Pseudomonas*, which were in decreased proportion in our findings [25]. The oral microbiome in smokers had *Bacteroides* as the most abundant bacterial genus, similar to our findings, though many other bacterial genera that were different from our study were also detected [21]. CSE exposure impacted the lung microbiota by increasing the proportion of Bacteroidetes (38.4%) and decreasing the proportion of Proteobacteria (26.2%) at the phylum level, and causing an increase in the genera *Bacteroides*, *Clostridiales, Shewanella*, *S24-7* and a decrease in the genera *Pseudomonas*, *Alkalibacterium, Acinetobacter*, *Mycoplasma*, *Oscillospira*, *Allobaculum*. In contrast to lung microbiota, the oral and nasal microbiota showed increased Firmicutes and decreased Proteobacteria in the CSE-exposed mice compared to the control mice. This is quite different from the changes observed in the oral microbiota of human smokers, where *Fusobacteria*, *Prevotella*, and *Selenomonas* were the most abundant genera, in contrast to *Peptococcus* and *Capnocytophaga* in non-smokers [21]. In contrast to the human oral microbiome, Bacteroidetes constituted only 7.6% and 10.46% of the total oral and nasal microbiota in the CSE-exposed mice in this study. In comparison, in the control mice, Proteobacteria was the major phylum constituting > 99% of the total oral and nasal microbiota. The quantitative differences between the human smokers’ data and our mice observations could be due to the use of smoke extract instead of inhaled smoke, which contains both intact particulates and chemical constituents.

### 4.2. CNT- and/or CSE-Induced Lung Damage and Pathology

Mucin protein is required for inhibiting microbial adherence to epithelial cells, mucosal lubrication, and surface tension reduction in the lung. Both CNT and CSE exposures had downregulating effects on *MUC5B* gene expression, although the co-exposure showed no significant effect (Figure 5a). This contrasts with earlier findings [46], which reported an upregulation of the *MUC5B* gene in mice lungs after receiving a total of four doses (36–109 µg/mouse) of MWCNT at 1-week intervals for 90 days. This upregulation may be attributed to the onset of a reparative mechanism during their long-term (90 days) exposure. An upregulation of *MUC5B* has been found to be linked with pulmonary interstitial lung fibrosis and COPD [47,48]. The downregulation of *MUC5B* in our study may be due to the sloughing of goblet cells. Furthermore, exposure to CNT or CSE has been linked with poor pathogen clearance (fungal and bacterial) in the lungs, which might be in part due to inhibition of mucus secretion [49,50]. In this context, surfactant protein A (SP-A), another important innate defense protein in the lungs, is required for the opsonization of pathogens for phagocytosis by the alveolar macrophages. We observed a significant decrease in SP-A expression both in CNT and CSE individual exposures and co-exposure, suggesting an increased propensity for infection in the exposed lungs (Figure 5b). Downregulation of SP-A has been reported to confer increased susceptibility to Streptococcal and Pseudomonas infections [51,52]. In contrast, the total Immunoglobin-A(IgA) level in BAL fluid was higher in the CNT and CSE exposure groups compared to the vehicle control group, implying upregulation of the mucosal secretory IgA (Figure 5d), which is required for the lung microbiota homeostasis and mucosal protection against infections. For instance, a decreased serum IgA level was associated with increased oropharyngeal colonization of microbiota members *Prevotella*, *Alloprevotella*, and *Selenomonas*, and severe lung disease [27].

### 4.3. Modulatory Effects of Lung Microbiota on Mucosal Homeostasis and Damage in Toxicant-Exposed Lungs

Our results showed a significant difference (either upregulation or downregulation) between microbiota-intact (normal) mice and microbiota-depleted (ABX) mice for induction of lung homeostasis and mucosal defense proteins (Figure 5), and pathological endpoints (Figure 6) in the toxicant-exposed lungs. The difference was more or less consistent in the three exposure types (CNT, CSE, or co-exposure), albeit with quantitative differences. Generally speaking, the lung innate homeostasis/mucosal defense protein MUC5B was upregulated whereas SP-A and AQP-1 were downregulated in microbiota-depleted mice compared to normal mice in all three exposure groups except for AQP-1 in the ABX-CSE background (Figure 5c). Collectively, this suggested the role of microbiota in maintaining lung homeostasis. Interestingly, we observed an abundance of short-chain fatty acid (SCFA)-producing bacterial phyla Bacteroidetes and Firmicutes. In this context, mucin metabolism produces SCFA, which preferentially serves as an energy source for the epithelial cells, reduces inflammation, and enhances the expression of epithelial tight junction proteins [53]. An earlier study associated *MUC5B* gene deficiency with colonization of *Streptococcus* and *Staphylococcus aureus*, increased accumulation of apoptotic macrophages, decreased phagocytosis, and reduced IL-23 in lungs [54]. Mucus is also required as an energy source by the commensal bacterial species *Akkermansia muciniphilla*, which promotes Treg differentiation and reduces inflammation and apoptosis in the epithelium [55]. Aquaporins contribute to the water permeability of the cell membranes, gas exchange, cell proliferation, and migration. Increased AQP-1 expression has been associated with angiogenesis and lung adenocarcinoma [56].

Taken together, the results suggested a differential (mediator-specific) modulatory effect of microbiota on mucosal proteins that are critical in lung innate defense and injury protection mechanisms in the exposed lungs.

Despite the demonstration of the hypothesized modulatory/causal role of oro-respiratory microbiota dysbiosis in lung mucosal toxicity/immunotoxicity due to nanoparticle exposures, our study had certain limitations. For instance, because the microbiota modulates the immune response via its immunoreactive metabolites or proteins, there is a need to identify specific molecular constituents driving the modulatory effect. Also, there is a need to further clarify whether the observed modulatory effect was directly mediated by the microbiota or indirectly via the activated immune mediators. Further research in this direction will help clear up this conundrum.

## 5. Conclusions

The current study, based on a sub-chronic exposure mouse model is the first to show that respiratory exposure to CNT or CSE, or their combination, causes significant dysbiosis of oro-respiratory microbiota, leading to perturbations in microbial composition, diversity, and species abundance in oral, nasal, and lung mucosal surfaces. Microbiota-depletion experiments demonstrated the causal role of oro-respiratory microbiota in modulating the innate defense and protective mechanisms against lung damage/pathology phenotypes induced in the CNT/CSE-exposed/co-exposed lungs; the modulatory effect showed a mediator-specific trend and varied with the type of exposure. Taken together, the study revealed the perturbations and importance of oro-respiratory mucosal microbiota in respiratory exposure to carbon nanoparticles, alone or in co-exposure with tobacco smoke. Our future efforts will be directed toward further understanding of the underlying molecular mechanisms of interaction between the lung microbiota and nanotoxicant responses.

## Data Availability

The data presented in this study are available on request from the corresponding author.

## Supplementary Materials

The following supporting information can be downloaded at: https://www.mdpi.com/article/10.3390/nano14030314/s1. Table S1. List of the primers used in the current study. References [5,26,57] are cited in the Supplementary Materials.
