# Supplementary material for: Oro-Respiratory Dysbiosis and Its Modulatory Effect on Lung Mucosal Toxicity during Exposure or Co-Exposure to Carbon Nanotubes and Cigarette Smoke
Source: Nanomaterials (Basel). 2024 Feb 4;14(3):314. doi: 10.3390/nano14030314 (PMC10856953; doi:10.3390/nano14030314)
Supplement: Supplementary file 1 [file nanomaterials-14-00314-s001.zip › nanomaterials-2796104-supplementary.pdf]

Supplementary Table S1. List of the primers used in the current study

Target gene	Primer	5' to 3' nucleotide sequence	Ta (⁰C)	Amplicon size (bp)	Reference
Hprt	F	TCC CAG CGT CGT GAT TAG C	60	169	[5]
	R	GTG ATG GCC TCC CAT CTC CT			
Muc5b	F	GAG GTC AAC ATC ACC TTC TGC	59.5	319	[26]
	R	TCT CAT GGT CAG TTG TGC AGG			
SP-A	F	GTA TTC TCG GCT GTA CCT GCC	60	300	[26]
	R	GAG GTC CAG GGT CTC CTT TGA			
AQP-1	F	CTG GCC TTT GGT TTG AGC AT	58	151	[26]
	R	CCA CAC ACT GGG CGA TGA T			
16S RNA	F	GTG CCA GCM GCC GCG GTA A	50	~300	[57]
	R	GGA CTA CHV GGG TWT CTA AT			

* Abbreviations: Annealing temperature (Ta); F and R represent 'Forward' primer and Reverse' primer, respectively.

Citations:

5. Frank, E.A.; Carreira, V.S.; Birch, M.E.; Yadav, J.S. Carbon Nanotube and Asbestos Exposures Induce Overlapping but Distinct Profiles of Lung Pathology in Non-Swiss Albino CF-1 Mice. *Toxicol. Pathol.* **2016**, *44*, 211–225. https://doi.org/10.1177/0192623315620587.
26. Bhattacharya, S.S.; Yadav, B.; Yadav, E.; Hus, A.; Yadav, N.; Kaur, P.; Rosen, L.; Jandarov, R.; Yadav, J.S. Differential Modulation of Lung Aquaporins among Other Pathophysiological Markers in Acute (Cl2 Gas) and Chronic (Carbon Nanoparticles, Cigarette Smoke) Respiratory Toxicity Mouse Models. *Front. Physiol.* **2022**, *13*, 880815. https://doi.org/10.3389/fphys.2022.880815.
57. Caporaso, J.G.; Lauber, C.L.; Walters, W.A.; Berg-Lyons, D.; Huntley, J.; Fierer, N.; Owens, S.M.; Betley, J.; Fraser, L.; Bauer, M.; et al. Ultra-high-throughput microbial community analysis on the Illumina HiSeq and MiSeq platforms. *ISME J.* 2012, 6, 1621–1624.
